# Intestinal Intussusception Due to Entrapped Ascaris lumbricoides in a 13-Year-Old Male Patient

**DOI:** 10.7759/cureus.33909

**Published:** 2023-01-18

**Authors:** Mohammad Nasim Khan, Isra Khan, Emad Alvi, Iftikhar Ahmad

**Affiliations:** 1 Department of Pediatrics, Umm Al-Qura University, Makkah, SAU; 2 Department of Radiology, Jawaharlal Nehru Medical College - Aligarh Muslim University, Aligarh, IND; 3 Department of Surgery, Jawaharlal Nehru Medical College - Aligarh Muslim University, Aligarh, IND

**Keywords:** ultrasonography, ascaris lumbricoides, intussusception, ileocolic, intestinal obstruction

## Abstract

*Ascaris lumbricoides* is a fairly common intestinal nematode affecting children worldwide, leading to major medical and surgical complications. Although most of the cases are asymptomatic, heavy infestation causes various acute abdominal complications. *Ascaris*-induced intestinal intussusception is one of the rare presentations.

We report a case of a 13-year-old boy with *Ascaris lumbricoides* infestation presenting with ileocolic intussusception. The patient presented to the emergency unit with colicky abdominal pain, vomiting, and constipation for two days. He was sick-looking and dehydrated. Further examination revealed diffuse abdominal distension with tenderness, guarding, and palpable mass in the right lower quadrant. Ultrasonography showed long-segment ileocolic intussusception with several worms in the intestinal lumen and adjacent mesenteric lymphadenopathy. An exploratory laparotomy was performed, which revealed ileocolic intussusception. The telescopic loop of the ileum was found to be gangrenous and was resected, and a loop ileostomy was performed. The patient was discharged on the seventh day postoperatively without any complications.

Physicians in tropical and subtropical countries should consider this condition in the differential diagnosis when they encounter similar presentations in their clinical practice. Sonography is a non-invasive, easy-to-use, and widely available imaging modality that can be employed to diagnose entrapped *Ascaris* in cases presenting with acute gastrointestinal complications. Early diagnosis and prompt surgical intervention can prevent bowel ischemia/gangrene and significantly reduce morbidity and mortality associated with such cases.

## Introduction

Ascariasis is an intestinal nematode caused by *Ascaris lumbricoides *(roundworm), affecting as many as 1.5 billion people worldwide; it is a significant cause of morbidity and mortality in various parts of the world [1]. It is quite common in tropical and subtropical regions, with the highest prevalence noted among children aged 2-10 years [2-3]. The mode of transmission is the fecal-oral route and risk factors include poor personal hygiene and sanitation, warm climate, and humidity [4].

While most of the infected children remain asymptomatic, a heavy parasitic infestation may lead to grave complications such as intestinal obstruction, volvulus, intussusception, and migration to other organs such as the biliary tract [5]. Most children with round worm-induced bowel obstructions respond to conservative management, and surgical management is reserved for complicated cases. Ultrasonography is a non-invasive, quick, and safe imaging modality that is helpful in the early diagnosis of intestinal intussusception and worm infestation, as it helps visualize entrapped *Ascaris lumbricoides* in the intestinal lumen.

This report documents a case of intussusception in a 13-year-old boy due to entrapped *Ascaris lumbricoides* in the intestinal lumen.

## Case presentation

A 13-year-old boy was brought to the emergency unit of the JN Medical College, Aligarh due to colicky abdominal pain, vomiting, and constipation for two days. Further history revealed four to five episodes of intermittent abdominal pain in the past six months. He had a history of pica and was not thriving well. Furthermore, he belonged to a farming family with an extremely low socioeconomic status.

On physical examination, he was sick-looking and moderately dehydrated with a temperature of 38 °C, pulse rate of 100 per minute, respiratory rate of 25 per minute, and BP of 110/70 mmHg. Abdominal examination revealed diffuse distension with tenderness, guarding, and palpable mass in the right lower quadrant of the abdomen. Routine investigations at the emergency unit revealed the following findings - hemoglobin (Hb): 8.8 g/dl, WBC count: 14000/mm^3^, neutrophils: 75%, eosinophils: 10%; the patient's platelet count was normal, with normal renal parameters and liver enzymes. Multiple air-fluid levels were detected on the erect abdominal radiograph, indicating intestinal obstruction.

**Figure 1:**
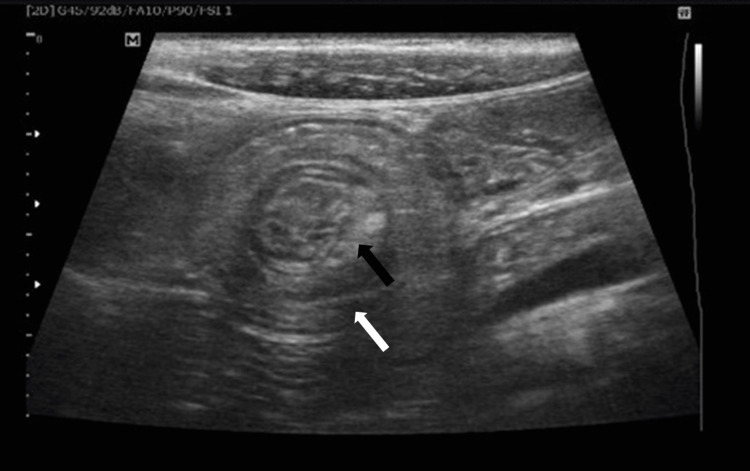
High-resolution transverse sonography of the abdomen via a linear transducer placed in the right iliac fossa region The image shows a swirled pattern of alternating hyperechogenicity and hypoechogenicity giving the classical "target" appearance representing the alternating layers of mucosa, muscularis, and serosa. Findings are consistent with intussusception with the inner bowel (black arrow) representing the intussuscipiens and the outer bowel (white arrow) representing the intussusceptum

**Figure 2:**
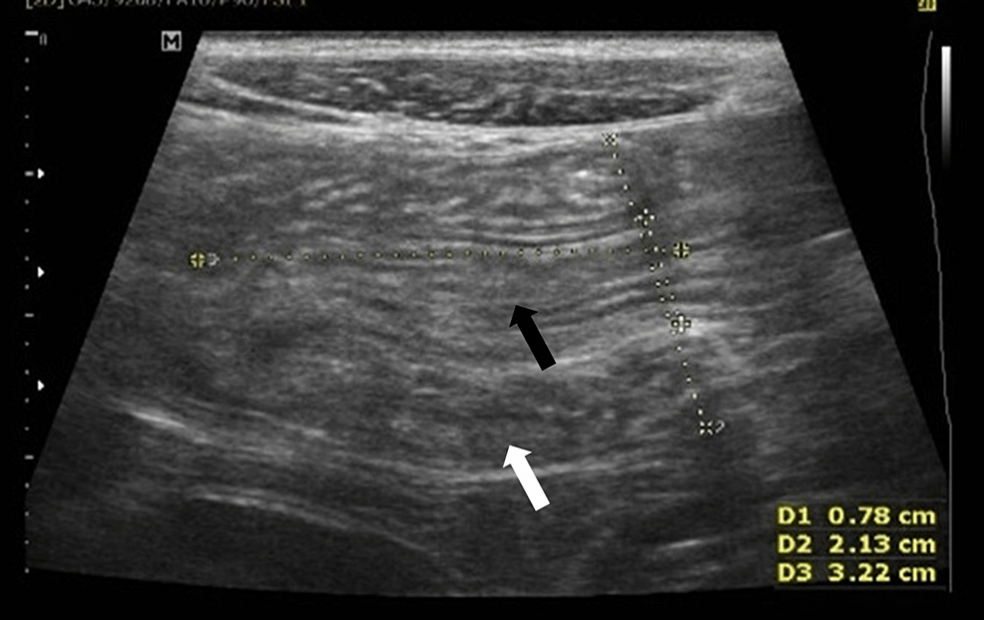
High-resolution sonographic view with a linear transducer placed in the right iliac fossa region in the longitudinal plane The image shows the telescoping of a segment of the small bowel loop (black arrow) into the large bowel (white arrow), suggestive of intussusception

**Figure 3:**
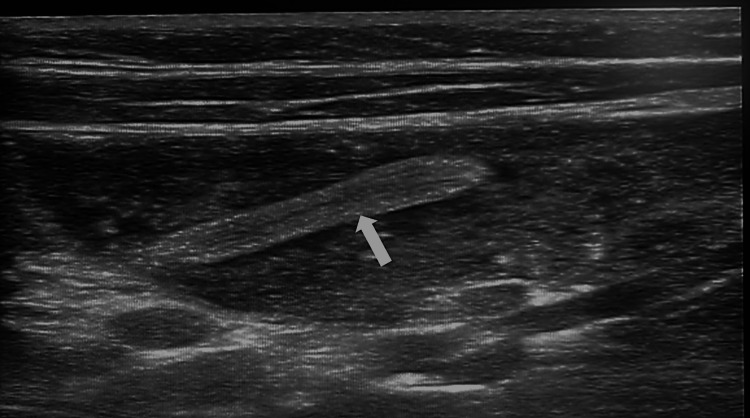
Proximal to the site of intussusception; the image shows a tubular hypoechoic structure with parallel echogenic lines in the dilated proximal bowel loop representing the characteristic appearance of an intestinal worm (white arrow)

**Figure 4:**
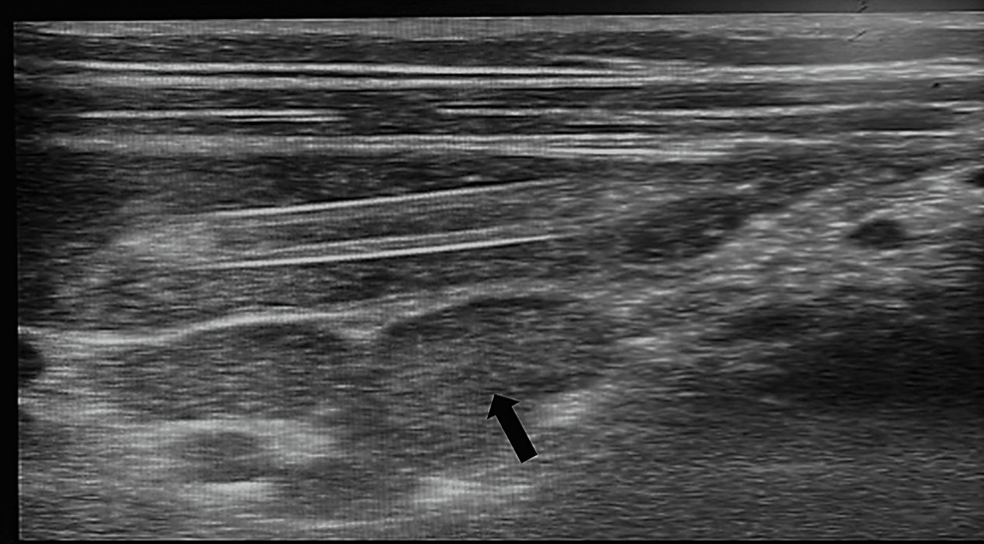
Multiple, rounded, and enlarged hypoechoic lymph nodes (black arrow) in adjacent mesentery suggestive of lymphadenopathy; however, no necrosis, calcification, or conglomeration was seen

High-resolution sonography on the transverse plane of the abdomen, via a linear transducer placed in the right iliac fossa region, showed a swirled pattern of alternating hyperechogenicity and hypoechogenicity. This gave the classical “target” appearance representing the alternating layers of mucosa, muscularis, and serosa. These findings were consistent with intussusception, with the inner bowel (Figure 1, black arrow) representing the intussuscipiens, and the outer bowel (Figure 1, white arrow) representing the intussusceptum. The longitudinal plane showed the telescoping of a segment of the small bowel loop (Figure 2, black arrow) into the large bowel (Figure 2, white arrow), suggestive of intussusception. As shown in Figure 3 and Figure 4, there was a tubular hypoechoic structure with parallel echogenic lines in the dilated proximal bowel loop, representing the characteristic appearance of the intestinal worm (white arrow), which suggests worm infestation. The images also showed multiple, rounded, and enlarged hypoechoic lymph nodes (black arrow) in the adjacent mesentery, suggestive of lymphadenopathy. However, no necrosis, calcification, or conglomeration was seen.

**Figure 5:**
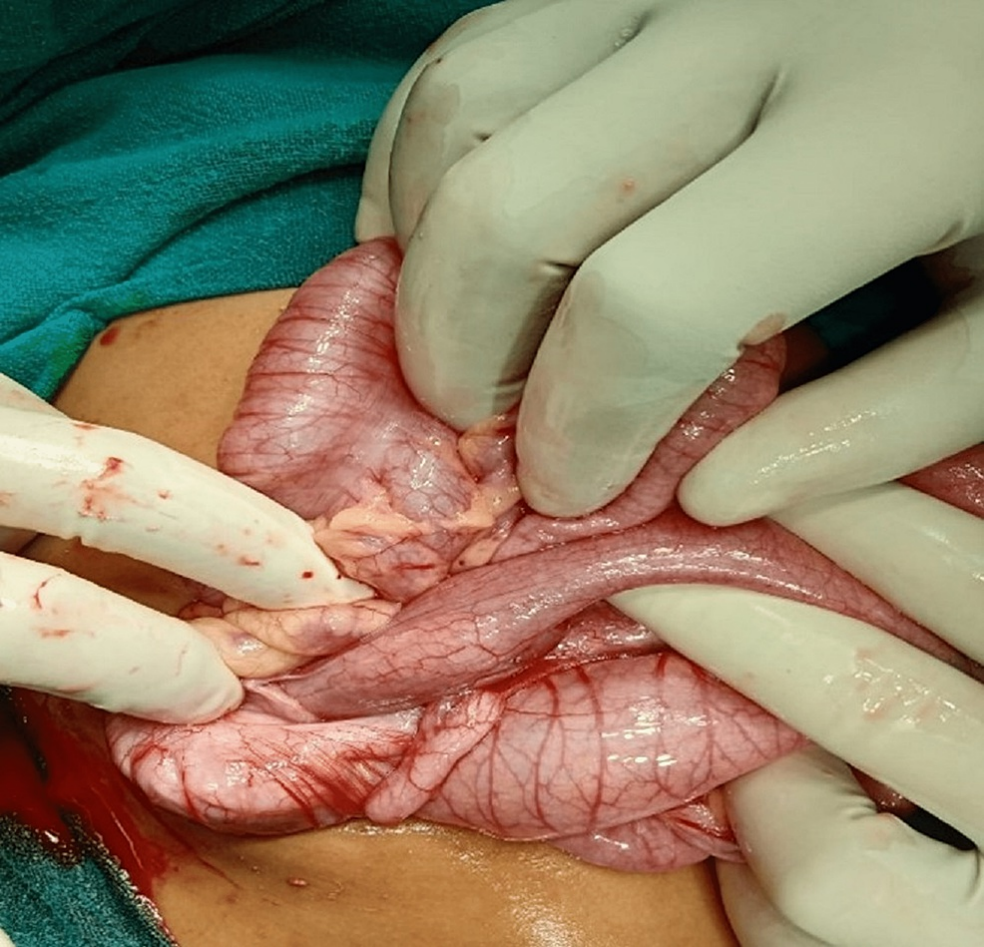
Intraoperative image showing the terminal ileum, appendix, and ileocolic junction

**Figure 6:**
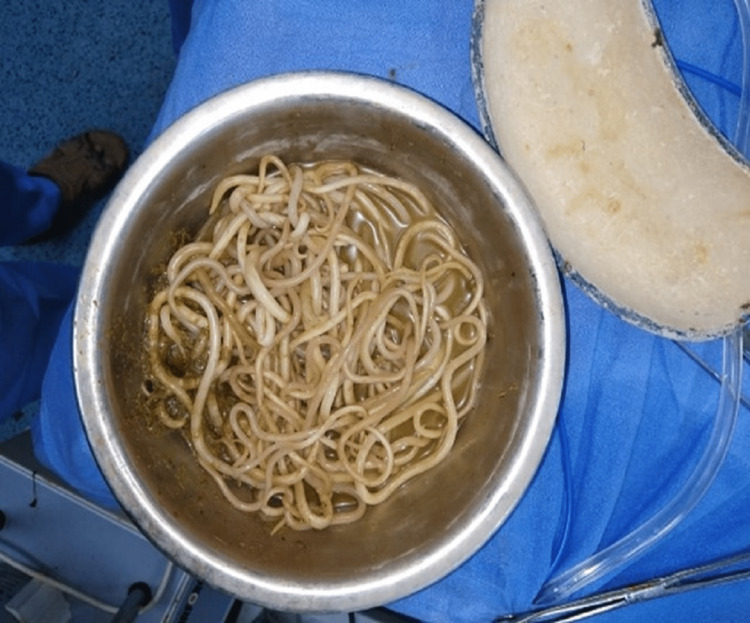
View of extracted Ascaris worms

Ultrasonography revealed a long-segment ileocolic intussusception, with associated intraluminal worms and adjacent lymphadenopathy. The child remained ill despite initial resuscitation and hence was given further IV fluid resuscitation, and antibiotics (IV cefotaxime, metronidazole) were initiated. An urgent laparotomy was planned at this time. Laparotomy revealed ileocolic intussusception. Figure 5 shows an intraoperative image of the terminal ileum, appendix, and ileocolic junction. The telescopic loop of the ileum was found to be gangrenous and was resected, and a loop ileostomy was performed. The clumped *Ascaris* was milked out intraoperatively (Figure 6).

Histopathological examination of the resected bowel segment revealed a varying degree of congestion, edema, and coagulative and hemorrhagic necrosis. Albendazole (400 mg OD for three days) was started. The rest of the worms subsequently came out from the stoma loop. The patient's condition improved significantly in the postoperative period and he was discharged on the seventh postoperative day, with the advice to undergo anastomosis and closure of ileostomy at a later stage. Deworming was done for the patient's family members who screened positive for ascariasis.

## Discussion

Ascariasis is one of the most prevalent nematode infestations, especially in tropical and subtropical regions where unhygienic disposal of human excreta is common, and it is transmitted via the fecal-oral route [4]. Repeated autoinfection allows the worms to multiply to overwhelming numbers, leading to a high parasitic load [6]. The majority of *Ascaris lumbricoides* infestations are asymptomatic. However, individuals with heavy worm infestation are likely to develop a wide spectrum of acute abdominal complications such as intestinal obstruction, biliary obstruction, cholangiohepatitis, pancreatitis, liver abscess, and acute appendicitis [7]. The other reported complications include severe gastrointestinal bleeding, perforation, and even gangrene of the bowel wall [8].

*Ascaris*-related intestinal obstruction is an acute problem in the developing world, with an incidence of 9.2 cases per 100,000 persons [9]. Children are particularly at risk of *Ascaris*-related intestinal obstruction due to the smaller size of the bowel lumen and often an increased parasitic load [5]. A host inflammatory reaction to worm-derived neurotoxins and anaphylatoxins may lead to small bowel inflammation and spasmodic contraction of the small bowel. This coupled with a bolus of *Ascaris* worms in the intestinal lumen at the ileocecal valve can cause intestinal obstruction [5]. If the mechanical obstacle at the ileocecal valve persists, the mass of *Ascaris* worms acts as a fixed point leading to intussusception or volvulus [5]. Most cases of intussusception (>90%) during childhood are idiopathic. A secondary cause constituting a lead point for intussusception is found only in 6-8% of the cases [10]. The most commonly encountered lead points are Meckel’s diverticulum, polyp, lymphoma, vascular malformation, intestinal worms, and Henoch-Schonlein purpura [10,11]. Intestinal intussusception caused by *Ascaris*, however, is very uncommon.

The histopathological study of the resected bowel segment in our case exhibited edema, congestion, and coagulative and hemorrhagic necrosis indicative of combined ischemia and venous outflow obstruction due to strangulation induced by intussusception. Plain radiographs, barium examinations, ultrasonography, and CT scan findings of *Ascaris lumbricoides* infestation and associated complications have been described in the literature [5,12].

The role of ultrasonography is of key importance in the early diagnosis of *Ascaris* mass in the intestinal lumen. The appearance of *Ascaris* as a long echogenic structure (linear or curved, single or multiple) with or without a central anechoic tube and mostly without acoustic shadowing on ultrasound has been reported in various studies in the literature [12-14]. Varied appearances like “winding highway” or parallel lines, “railway track” signs on longitudinal scans, doughnut or target signs, and ring sign bull’s eye appearance on transverse section have also been described [12,14,15].

The management of uncomplicated ascariasis is usually conservative and involves anti-parasitic drugs such as albendazole, mebendazole, or pyrantel pamoate. Partial intestinal obstruction due to ascariasis may resolve spontaneously with bowel rest, nasogastric decompression, anthelminthic treatment, and fluid-electrolyte replacement [16]. In more critical cases of intestinal obstruction/perforation/peritonitis/, various surgical interventions are required, such as the resection of the gangrenous bowel with primary anastomosis, enterotomy, and milking of the obstructing worms in the caecum or colon [17]. Our patient was managed surgically, with procedures consisting of intussusception relief, resection of the gangrenous ileum, and ileostomy.

A high index of suspicion, early diagnosis, and prompt intervention can prevent bowel ischemia/gangrene and significantly reduce morbidity and mortality associated with such cases. Primary prevention of ascariasis involves maintaining proper hygiene and sanitation practices, and periodic deworming on detection of ova/cysts on microscopic stool analysis [6].

## Conclusions

Acute intestinal obstruction is one of the major causes of morbidity and mortality in children in developing countries. Intestinal intussusception requires early diagnosis, as it usually constitutes a strangulation/obstruction type of intestinal obstruction that quickly leads to bowel ischemia and gangrene and can cause high mortality. Ultrasonography is a very useful diagnostic tool for the early detection of these complications and for localizing balls of *Ascaris* worms in the intestinal lumen. Information/guidelines to raise awareness about ascariasis and its preventive measures should be included in all health education programs and should be communicated to school children and their parents/caregivers to prevent the risk of infection.
